# Yaws active case detection surveys in 15 districts of Cote d’Ivoire

**DOI:** 10.1371/journal.pone.0348510

**Published:** 2026-05-28

**Authors:** Aboa Paul Koffi, Didier Yao Koffi, Elysée Gobe Agodio, Théodore Yao, N’cho Mouroh Chantal N’guessan, Amari Jules Cesar Akpa, Eguzkiñe Muñoz Trevilla, Notie Berté, N’zebo N’goran, Nzrasse Florent Niamke, Agui Sylvetre Dizoe, N’goran Raphael N’dri, Priya Pathak, Beshah Abaté Mulugeta, Yves Thierry Barogui, Kingsley Bampoe Asiedu, Mamadou Kaloga

**Affiliations:** 1 Programme National de Lutte contre l’ulcère de Buruli et des Maladies cutanées ulcératives endémiques, Abidjan, Côte d’Ivoire; 2 Unité de formation et de recherche en biosciences, Université Félix Houphouët-Boigny, Abidjan, Côte d’Ivoire; 3 Centre Suisse de Recherches Scientifiques en Côte d’Ivoire, Abidjan, Côte d’Ivoire; 4 Unité de formation en géographie, Université Félix Houphouët-Boigny, Abidjan, Côte d’Ivoire; 5 Fondation Anesvad, Bilbao, Spain; 6 Programme National d’Elimination de la Lèpre de Côte d’ivoire, Abidjan, Côte d’Ivoire; 7 World Health Organization, country office, Abidjan, Côte d’Ivoire; 8 World Health Organization, Headquarters, Geneva, Switzerland; 9 World Health Organization, Regional Office for Africa, Brazzaville, Congo; 10 Unité de formation et de recherche en sciences Médicales, Université Félix Houphouët-Boigny, Abidjan, Côte d’Ivoire; GGD Amsterdam, NETHERLANDS, KINGDOM OF THE

## Abstract

**Background:**

Yaws is one of the skin-related neglected tropical disease (skin NTD). It is closely linked to poverty and occurs mainly in poor communities in hot, humid, tropical regions. Children in disadvantaged communities in the tropics are most affected. Following the recommendations of the WHO NTD Road map 2021–2030, Côte d’Ivoire planned an evaluation (clinical and serological investigation) to assess the true extent of yaws in the country. Côte d’Ivoire is one of the nine countries currently endemic for yaws in the WHO African Region. The aim of this investigation was to establish the endemicity of yaws and to gather information that will inform planning for yaws eradication in Côte d’Ivoire.

**Method:**

A public health intervention involving active case-finding was conducted in 345 health areas of 15 targeted health districts. All children aged 2–15 years in village primary schools and in households were systematically recruited and examined. All enrolled children with suspicious lesions were initially tested with Standard Diagnostic (SD) serological treponemal test. Cases positive for treponemal SD test were subsequently tested using the dual treponemal and non-treponemal point-of-care test (DPP), a rapid serological test that is used to confirm yaws. All confirmed cases were treated with a single dose of azithromycin according to age. Additionally, other skin NTD and skin conditions were detected.

**Results:**

A total of 3870 villages and hamlets were investigated in which 486 975 children were targeted. Overall, 65 918 (14.66%) children with various skin lesions were identified. Two hundred and ninety-one (291) RDT (Rapid Diagnostic Test) positive cases of yaws were documented. Half of the RDT positive cases (146/291) were confirmed as DPP positive. Children aged 5–14 years (*n* = 129) represented 88% of DPP positive cases. The four most common skin conditions identified were traumatic wounds (13 855; 21.07%), dermatophytosis (15 640; 23.8%), pityriasis versicolor (6998; 10.64%) and scabies (5955; 9.05%).

**Conclusion:**

This rapid investigation confirmed the endemicity of yaws in Côte d’Ivoire at low and variable prevalence. Treatment interventions will be adapted to the level of endemicity that we have found. Analysis of the results opens avenues of research to better understand the endemicity of yaws in Côte d’Ivoire.

## Introduction

Yaws, also known as *Frambœsia tropica*, is a non-venereal skin-related neglected tropical disease (skin NTD) [1,2] caused by infection with *Treponema pallidum* subspecies *pertenue*. Its spread is facilitated by overcrowding and poor hygienic conditions [3]. Among the endemic trepomenatoses (yaws, bejel and pinta), yaws is the most common and also the most damaging, causing skin complications and osteoarticular damage (bone deformation, mutilation, destruction of the nasal pyramid and chronic disability) [4]. It can also cause pain and skin induration, with or without inflammation, in bones and joints [4]. Yaws is closely linked to poverty and is most prevalent in poor communities in hot, humid and tropical regions of Africa, Asia, West Pacific and Latin America [5]. Nearly 75% of children aged under 15 years in disadvantaged communities in the tropics are at risk of developing yaws; incidence peaks in children aged 6–10 years [6]. Yaws has been known in Côte d’Ivoire since the 1950s. Between 1950 and 1960, the WHO and UNICEF developed and implemented a mass treatment program that led to a significant decline in the disease (Fitzpatrick *et al.*, 2014). In recent years, however, the disease has re-emerged. Today, yaws is still considered endemic in Côte d’Ivoire, based on recent health data. Annual Report on Health Statistics (ARHS 2000). Following the recommendations of the WHO road map on NTDs 2021–2030 (the road map) [7], Côte d’Ivoire joined the eradication process and conducted a rapid epidemiological evaluation (clinical and serological investigation) to gain insights into the endemicity of yaws in the study areas.

Because we did not have enough resources to investigate all 113 districts, we selected 15 districts that reflect all major regions of the country and have varying levels of yaws based on the Annual Report on Health Statistics (Côte d’Ivoire Annual Report on the Health Situation | GHDx (healthdata.org) for yaws on routinely reported, clinically suspected cases. Districts were classified into three criteria: districts with cases of low, medium and high suspicion of yaws. These yaws data are based solely on the presence of yaws-like lesions without any serological testing. Thus, 15 districts were identified considering the climatic conditions and socio-cultural characteristics of the country. The data generated will be used to plan therapeutic interventions in communities. The investigation was carried out according to the guidelines that WHO defined at a meeting in Abidjan (May 2018), after which the experts proposed investigating districts in the country centripetally from the most remote villages to the health centre. This action is part of the first stage of the WHO yaws eradication strategy (2018).

This study aims to determine the endemicity of yaws and to generate a distribution map in accordance with the WHO roadmap, with the ultimate goal of informing strategies for the eradication of yaws in Côte d’Ivoire. This involved capacity-building of district management teams, subdistrict nurses, community health workers (CHWs) and teachers to support the investigation.

## 1. Method

### 1.1 Investigation design

The study was designed as a public health intervention. It was a descriptive cross-sectional survey with integrated active case detection, conducted in villages and hamlets with poor access to health care, where population activities are based on livestock production and subsistence farming. These communities have inadequate sanitation and potable water, with limited accessibility to health services. All the rural communities in the health districts targeted by this study were considered. The health area was the unit of implementation, and the endemicity of the district was defined according to the health area. The investigation was carried out centripetally from the most remote villages to the health centre, examining all children in and out of school in the households. Skin NTDs other than yaws, such as Buruli ulcer, leprosy and scabies, were also screened for as well as other skin conditions.

### 1.2 Study sites and time period

The integrated approach was adopted in 15 selected districts in five zones in Côte d’Ivoire in conjunction with the National Buruli Ulcer and Yaws Control Programme and the Leprosy Control Programme of Côte d’Ivoire. The selected districts, located in the southern east and west, the middle and the northern east and west zones, were: Abengourou, Adiaké, Agboville, Bangolo, Bondoukou, Didievi, Issia, Korhogo, Minignan, Nassian, Tanda, Tiebissou, Touba, Vavoua and Sassandra.

The five zones have different vegetation and rainfall seasons, as well as socio-cultural realities. The north is more arid, with low rainfall throughout the year; its vegetation is dominated by savannah, and population density per square kilometer is lower than in the southern part of the country [8]. The centre of the country is an area of wooded savannah with average rainfall, straddling the more arid northern zone and the forest zone to the south. Three districts are targeted in each of the five zones. Districts included in the study were selected based on the number of suspected cases reported in ARHS. In each zone, the districts were divided according to three criteria: the district reporting no cases or very few cases (1–500 cases); the district reporting a moderate number of suspected cases (100–1000 cases); and the district reporting the highest number of suspected cases (500–1000 cases).

The survey was carried out according to the school calendar so that as many pupils as possible could be enrolled. The survey took place in three stages: November to December 2021; November to December 2022; and February to March 2023.

### 1.3 Target population

It was a total population sampling of children. All children aged 2–15 years were systematically recruited. As yaws often appears in clusters, such as schools or hamlets [9], participants were mainly recruited in village primary schools and households, and in two secondary schools. Individuals over 15 years of age were excluded from the yaws screening study, it’s prevalence is mostly for children under the age of 15 years [10]. However, any individual (adult or child) presenting with non-yaws-like lesions was screened for Buruli ulcer, leprosy, scabies, or other common skin conditions.

### 1.4 Implementation

Implementation steps included preparatory meetings with stakeholders, training of health workers and CHWs, integrated case searches, serological diagnostic tests, laboratory confirmation and reporting, patient management, and monitoring and evaluation visits. Integrated case searches, diagnosis and management happened concurrently.

#### 1.4.1 Stakeholder meetings.

The stakeholder meetings were attended by representatives from the Ministry of Health (including the control programmes, district health authorities and head of health facilities), the Pasteur Institute for laboratory issues, district and community leaders and representatives from the Anesvad Foundation (Bilbao, Spain). In each district, meetings were also held with the district health directorate, local and traditional authorities as well as school administrators to introduce the project and seek their support for its implementation.

#### 1.4.2 Training.

The training participants were carefully selected in consultation with the districts and in collaboration with the national programme managers. The categories of staff trained included medical doctors, public health nurses, laboratory technicians, CHWs and selected teachers. The training included information on disease epidemiology, clinical recognition and diagnosis, differential diagnosis, sample collection and transport, social consequences of skin NTDs, reporting tools and disease management. The content of the training was adapted to the cadre receiving the training. For CHWs and teachers, training focused mainly on clinical recognition (skin and bone lesions) to increase their ability to suspect and appropriately link cases to the health-care system. Similarly, for health workers, the focus was on clinical diagnosis, sample collection and transport, rapid diagnostic tests for yaws and management of confirmed cases. On-site training workshops were organized in each district using the harmonized standard operating procedures [11].

#### 1.4.3 Integrated active case-finding.

Active case searches were conducted in the 15 districts according to a schedule established by local health authorities, including visits to all villages and primary schools in rural areas. WHO posters and booklets on Buruli ulcer, leprosy, and yaws were used as screening tools. Children not attending school were registered through door-to-door visits by trained community health workers, while those attending school were registered by teachers.

#### 1.4.4 Clinical examination and serological diagnostic test.

Suspected cases of yaws, leprosy, Buruli ulcer, and scabies were examined. All children aged 2–15 years and adolescents aged 16 years and above presenting with skin lesions were invited to report to the clinical teams. Two consultation rooms were organized: one for adults with other skin conditions and another for detailed assessment of suspected lesions in children following a triage process.

Triage was conducted by trained community health workers, who separated individuals with visible skin lesions from those without. For greater diagnostic certainty, clothing was removed when necessary to allow full skin examination. Clinical assessments were then performed by nurses with support from the district NTD focal point.

In addition, a WhatsApp platform was established to allow nurses to share photographs of suspected lesions for remote consultation with three dermatologists in Abidjan and physicians at the central level.

#### 1.4.5 Serological confirmation tools.

**Fig 1:**
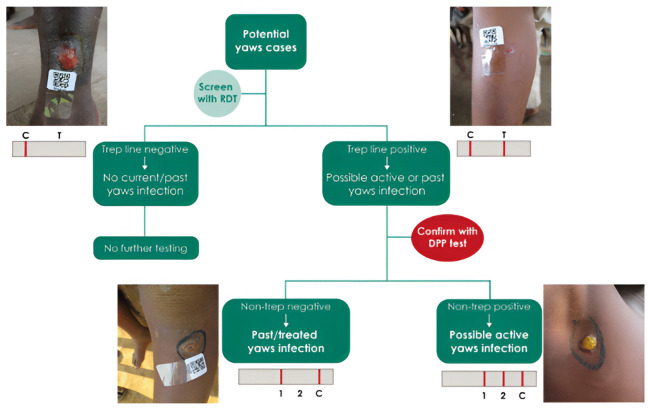
Flow chart for the sequential use of rapid treponemal and DPP tests for yaws [6].

Two tests were carried out in the field. The first test was the Standard Diagnostic (SD) syphilis 3.0 (multi) RDT test manufactured by Standard Diagnostic in Germany (Fig 1) which was systematically performed on enrolled children with lesions. This RDT is positive in people with a prior history of *Treponema pallidum* infection, regardless of current infection status.

The diagnostic protocol followed the sequential system recommended by the World Health Organization for yaws eradication, which involves the use of rapid treponemal and DPP tests [6]

The second test used was the Chembio DPP (Dual Path Platform) Syphilis Screen & Confirm kit [12], a rapid serological test designed to detect both treponemal and non-treponemal antibodies. This test served as a confirmatory tool for cases that tested positive with the SD test. The DPP is a dual-entry platform containing both treponemal and non-treponemal antibodies. Its positivity, determined by three lines, confirms the lesion as a case of yaws (Fig 1).

The DPP micro-reader was used to detect faint positive lines not easily visible to the naked eye. It provides qualitative results (positive/negative) and quantitative measurements of treponemal infection intensity. Although the manufacturer defines positivity at a cut-off ≥9 (1:1), this threshold is very low. Therefore, cut-offs of 12 (1:2) and 24 (1:4) were used for the analysis. [12].

Only suspected cases of yaws serologically confirmed by DPP were recorded as yaws cases (Fig 1).

#### 1.4.6 Patient care.

Patients were treated according to the disease. For yaws, patients receive a single dose of azithromycin. Yaws patients contacts were not treated; this was not the standard of care in this setting and was not performed as part of this activity. Leprosy patients were treated at various districts as per WHO recommendations. Patients with other wounds and skin conditions were treated onsite and then referred to the nearest health facility for further management.

#### 1.4.7 Mapping.

GPS coordinates were taken by trained field staff with smartphones in the villages and hamlets where cases of yaws had been confirmed. Field staff was instructed to ask yaws cases their usual residency, to distinguish between autochthonous cases and imported cases.

#### 1.4.8 Monitoring and supervision.

To maintain the skills and knowledge of the trained health personnel, a monitoring and supervisory system was instituted in all 15 districts. During the field visits, personnel skills and adherence to protocols, clinical examination, documentation, storage was evaluated. A monitoring tool which was a checklist for controlling compliance with the investigation implementation protocol was used. It checked the enrolment of children, yaws test performance, antibiotic administration and resistance, other skin conditions diagnostic and management. Feedback was provided for the district staff. Supervision was undertaken by Buruli ulcer, yaws and leprosy control program staff, the Anesvad Foundation and WHO experts.

### 1.5 Data management

The data of each patient; age, gender, origin, test result, diagnostic, treatment, GPS coordinates were collected on form WHO/Yaws/003 and entered the district health information software (DHIS2) in real time by each district data manager. The data were analysed by the PNLUB responsible for yaws, with the support of WHO experts.

### 1.6 Regulatory parameters under which this work was performed

This study was conducted as a public health surveillance activity under the authority of the government of Cote d’Ivoire Ministry of Health, public hygiene and universal health coverage (number N°1390/MSPHPCMU/DGS/PNLUB/Kaf). The need for consent was waived.

## 2. Results

### 2.1 Geographical target reached

All 15 health districts were investigated by 345 teams who carried out the investigation in 345 health areas, achieving a health area coverage rate of 100%. This activity took place over 21 days in each district. In the villages and hamlets to be investigated, the coverage rate was 88% (3178 of 3621 sites) because the nurses could not reach some localities during the rainy season.

### 2.2 Target children reached

Of the total of 1 047 085 children aged from 2 to 15 years identified in the 15 health districts, 486 975 (46.5%) living in rural areas were targeted in this investigation. The triage carried out by CHWs reached 92.3% (*n* = 449 647) of the targeted children, of whom 65 918 had skin lesions (i.e., 14.7% of the children examined (Table 1).)

**Table 1:** Proportion of children with lesions by district investigated.

Health district	No. of children targeted (all districts)	No. of children targeted in health areas (rural area)	No. of children examined in rural areas	No. of children with skin lesions (%)
Issia	80 650	36 293	33 873	5 075 (14.9%)
Bangolo	100 769	45 346	42 323	5 591 (13.2%)
Sassandra	56 895	25 603	23 896	4 418 (18.5%)
Adiaké	30 521	13 734	12 818	4 119 (32.1%)
Abengourou	128 253	57 714	53 865	6 398 (11.9%)
Agboville	113 731	51 179	47 767	7 163 (15.0%)
Vavoua	172 481	77 616	72 441	6 077 (8.4%)
Tiebissou	41 809	18 814	17 559	5 696 (32.4%)
Didievi	40 958	18 431	17 202	4 316 (25.1%)
Bondoukou	130 921	58 914	53 023	5 282 (9.9%)
Touba	51 187	23 034	21 422	3 986 (18.6%)
Nassian	19 627	19 627	18 646	2 438 (13.1%)
Minignan	39 544	17 795	14 948	1 539 (10.3%)
Korhogo 2	9 077	9 077	7 170	1 219 (17.0%)
Tanda	30 662	13 798	12 694	2 601 (20.5%)
**Total**	**1 047 085**	**486 975**	**449 647**	**65 918 (14.7%)**

### 2.3 Clinico-serological results for yaws

Overall, 65 918 children with skin lesions underwent SD treponemal test, of whom 0.4% (*n* = 219) tested positive for SD treponemal test and 99.6% (65 627) tested negative for SD treponemal test.

The health districts of Agboville and Abengourou, located in the south-east of Côte d’Ivoire in the forest zone, had the highest rates of positive SD treponemal test, at 1.1% and 2.1% respectively. No positive RDTs were recorded in the Nassian and Touba health districts in the north-east and west of the country.

Among those tested with DPP, 146 (50.2%) were confirmed positive. Based on the number of children with skin lesions (*n* = 65 918), the rate of confirmed yaws is 0.22% (*n* = 146). However, based on the total rural children targeted for the screening (n = 486 975) yaws prevalence could be evaluated to 0.03% (n = 146).

Lesions were dominated by circular less painful ulcers on limbs followed by scaly macules and papillomatous, nodular yaws skin lesions. Agboville (0.49%) and Abengourou (1.3%) had the highest number of cases of DPP-confirmed yaws, followed by Issia (0.18%) and Sassandra (0.13%), all of which are in the forest zone (Table 2).

**Table 2:** Rates of RDT^*^ and DPP^#^ positivity by district of 2-15 years old children.

Health district	No. of skin lesions tested by RDT	No. of positive RDT (%)	No. of positive DPP (%)
Abengourou	6398	137 (2,14%)	82 (1.3%)
Agboville	7163	76 (1,06%)	35 (0.49%)
Issia	5591	21 (0.38%)	10 (0.18%)
Sassandra	4418	11 (0.25%)	6 (0.13%)
Bangolo	5075	3 (0.06%)	3 (0.06%)
Adiaké	4119	3 (0.07%)	2 (0.07%)
Didievi	4316	4 (0.09%)	2 (0.05%)
Vavoua	6077	5 (0.08%)	2 (0.03%)
Tanda	2 601	5 (0.19%)	2 (0.07%)
Minignan	1539	1 (0.06%)	1 (0.06%)
Tiébissou	5696	3 (0,05%)	1 (0.02%)
Bondoukou	5282	20 (0.38%)	0 (0.00%)
Touba	3986	0 (0.00%)	0 (0.00%)
Nassian	2438	0 (0.00%)	0 (0.00%)
Korhogo 2	1219	2 (0.16%)	0 (0.00%)
**Total**	**65 918**	**291 (0.44%)**	**146 (0.22%)**

**RDT**: Rapid Diagnostic Test, **DPP**: Dual Path Platform.

Azithromycin was used to treat positive cases of yaws in single doses according to age group.

**Fig 2:**
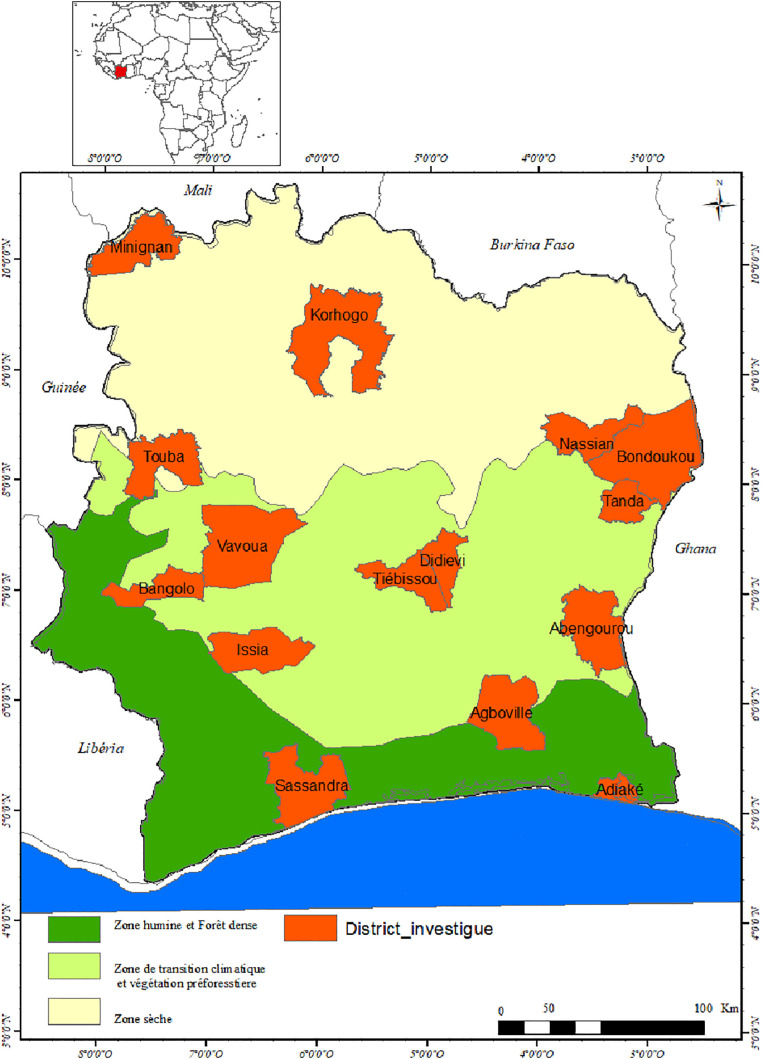
Positives cases of yaws in endemic localities.

Apart from Bangolo health district, where one of the three positive cases was an internal imported case from Divo health district, all the other positive cases in the other health districts were autochthonous cases (Fig 2).

**Fig 3:**
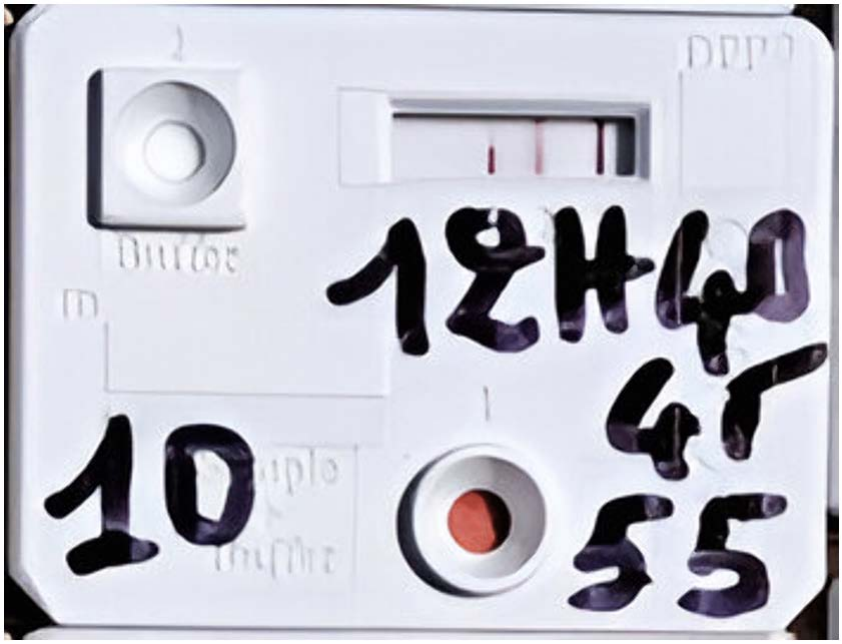
10 years old boy with yaws positive ulcerated skin lesion in Abengourou district in Côte d’Ivoire in 2022.

**Fig 4:**
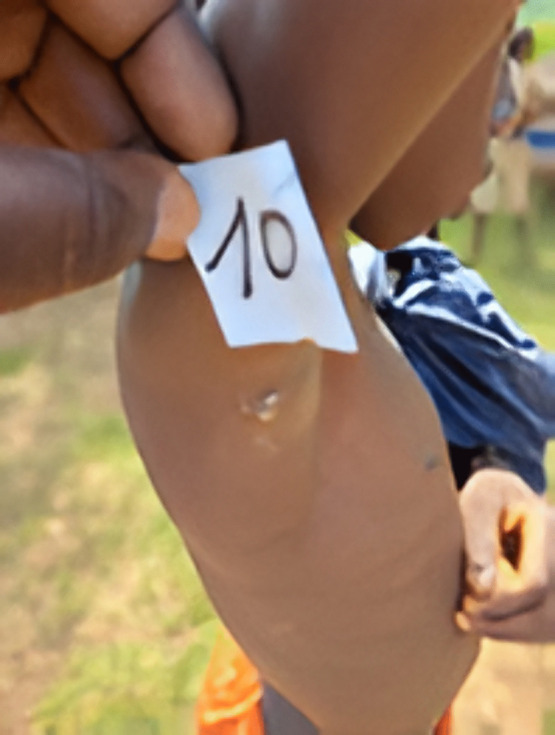
Positive DPP test of yaws lesion of a 10-year-old boy in Abengourou district in Côte d’Ivoire in 2022.

**Fig 5:**
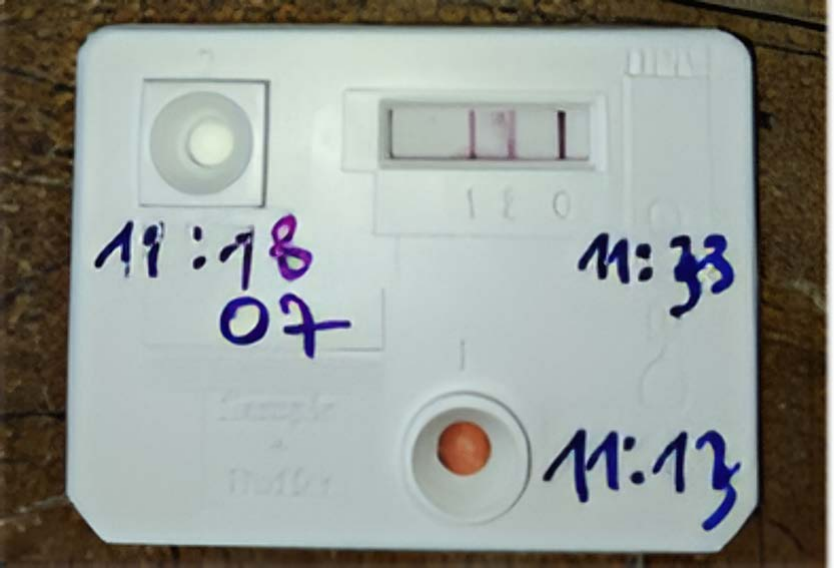
8 years old boy with yaws positive papillomatous, nodular skin lesions in Agboville district in Côte d’Ivoire in 2022.

**Fig 6:**
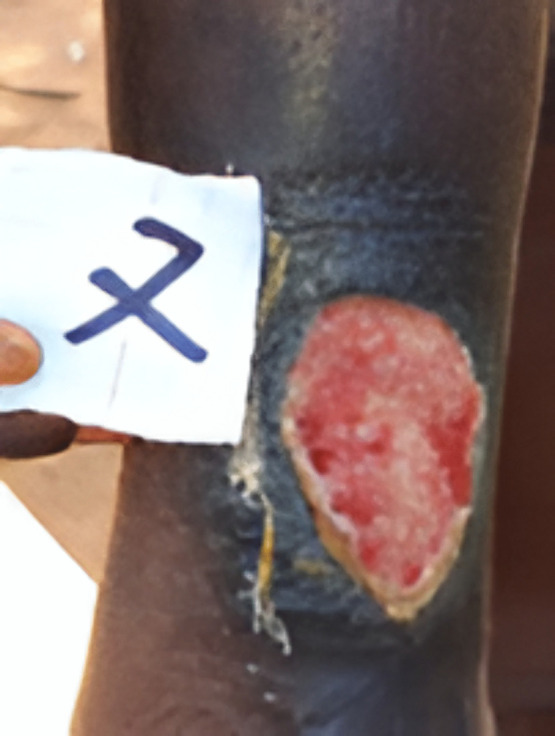
Positive DPP test of a 8-year-old boy in Agboville district in Côte d’Ivoire in 2022.

The positive cases came from 42 villages and hamlets in the districts investigated (Fig 3–6).

### 2.4 Socio-demographic characteristics of yaws cases

The results show that 94 of the confirmed cases were male and 52 females (i.e., a sex ratio of 1.81). Apart from Agboville health district, where 19 of the yaws cases were female (54.3%), in the other districts, males were the most affected by the disease.

The age group most affected is 5–10 years (*n* = 129) and represents 88% of cases in our study, the mean age is 9.03 years old, the median age is 8 years old with SD (3.09) and the range is from [2–15] (Table 3).

**Table 3:** Socio-demographic characteristics of yaws cases of 2-15 years old children.

Health districts with positives cases	Age (years) Mean (9.30), SD (3.09) Median (8) Range [2–15]			Sex (M^*^/F^#^)		Distance from health centre (km)			Origin of patient (for positives cases)	
	1^rst^	2^nd^	3^rd^	M	F	≤ 5	6–15	≥16	Autochthonous	Imported
1	13	39	30	54	28	82	0	0	82	0
2	0	13	22	16	19	35	0	0	35	0
3	3	5	2	9	1	10	0	0	10	0
4	1	3	2	4	2	6	0	0	6	0
5	0	3	0	2	1	0	3	0	2	1
6	0	2	0	2	0	2	0	0	2	0
7	0	2	0	2	0	2	0	0	2	0
8	0	1	1	2	0	2	0	0	2	0
9	0	1	1	1	1	2	0	0	2	0
10	0	0	1	1	0	1	0	0	1	0
11	0	1	0	1	0	1	0	0	1	0
**Total**	**17**	**70**	**59**	**94**	**52**	**143**	**3**	**0**	**145**	**1**

**M**: Male, **F**: Female.

The majority (97.94%) of patients live within a radius of 5 km of a health centre according to each district’s monograph, except for the three cases detected in health district 5 (Table 3).

### 2.5 Other skin diseases detected

The other skin lesions encountered in the field were reported on the WHO/Yaws/003 form when yaws tests were negative. A total of 65772 children and adults presented other skin diseases. Few NTDs, aside from scabies, were detected in all the skin lesions; no case of Buruli ulcer, only one case of leprosy, while 5955 cases of scabies were reported. Numerous other skin lesions were also reported in the communities. Table 4 gives a breakdown of the other skin lesion diagnoses reported. The most prevalent lesions in this study were dermatophytosis (23.8%) and traumatic wounds (21%).

**Table 4:** Skin lesions other than yaws detected in the target districts.

Non-yaws lesions	Number (%)
Dermatophytosis	15 640 (23.8%)
Traumatic wound	13 855 (21.1%)
Not specified	13 041 (19.8%)
pityriasis versicolor	6998 (10.6%)
Scabies	5955 (9.0%)
Lamellar ichthyosis	3357 (5.1%)
Molluscum contagiosum	3162 (4.8%)
Eczema	2320 (3.5%)
Post-erysipelas fasciitis	1443 (2.2%)
Leprosy	1 (0.0%)
Buruli ulcer	0 (0.0%)
**Total**	**65 772 (100.0%)**

## Discussion

The aim of the investigation was to establish the endemicity of yaws in Côte d’Ivoire. This investigation was carried out in 15 health districts using an integrated approach in line with WHO recommendations on NTDs [13]. It involved 345 health areas with 3178 villages and hamlets of 3621 initially targeted, giving a geographical coverage rate of 88%. Cases of yaws were confirmed using DPP tests. This is a standardized tool recommended by WHO for the eradication of yaws, as used by **Piamale** *et al.,* 2023 [14].

The investigation focused exclusively on rural areas because, as **Mitja** *et al,* 2015 [15] report, yaws occurs mainly in rural communities with limited access to health facilities. In all 15 districts, 46.5% of children live in rural areas. This proportion is slightly higher than the rate of children aged under 15 years living in rural areas reported by the 2021 general population census in Côte d’Ivoire (38.8%) (**EDS** 2021). Of these children, 92.3% were examined for skin lesions related to yaws during the study. CHWs played a crucial role in identifying children with skin lesions; this approach was also tried and tested by **Boock** *et al.,* 2017 [16] in Cameroon, where community case detection carried out in remote communities by hospital staff relied on CHWs to identify cases. The proportion of children with skin lesions found in our study (14.7%) is higher than that (8%) reported by Kazadi et al. 2014 [17] in a study carried out in the Central African Republic.

Suspected cases of yaws in our survey (0.44%) are much lower but could not be compared with those found by **Hassan** *et al.,* 2021 [18] in the context of screening for the re-emergence of yaws in Nigeria (64.4%). In their study the 64.4% suspected cases reported were based on clinical presentation alone, with only 3.7% later confirmed by serological tests (VDRL), unlike our study where all cases of skin lesions were investigated.

Incidence was not measured in the yaws surveillance. Among the villages and hamlets investigated in the targeted district, 42 were endemic. These 42 villages and hamlets were in 35 health areas of 11 districts.

This endemicity was based on the definition given in the study by **Basing** *et al.,* 2020 in Ghana, which stipulates that a community is endemic if at least one person has tested positive for DPP. In terms of health districts, the highest prevalence was found in Agboville (0.49%) and Abengourou (1.3%). Overall prevalence in the 15 districts is lower than that found by **Agana** *et al.,* 2024 [19] in a study of yaws prevalence in Ghana (1.92%). The districts with the highest prevalence of yaws in Côte d’Ivoire are in forest areas with high rainfall patterns, as indicated by the study by **Kouao** et al. 2020 [20] in an analysis of climatic regionalization in the context of changing climate. This result accords with that of **Kazadi** *et al.,* 2014 [17] who states that the highest prevalence of yaws in the tropics seems to be closely linked to high rainfall; and is confirmed in the health districts located in the dry regions of Côte d’Ivoire where only one case of yaws was recorded.

It appears that among the yaws cases diagnosed in our study, the male gender is dominant, a result concordant with that of **Boock** *et al.,* 2017 [16] where a greater number of male cases were found in all age groups in a study of yaws resurgence in Cameroon. **Kazadi** *et al.,* 2014 [17] also emphasize this finding in update of yaws epidemiology by saying that it appears that rather more males than females suffer from the disease. This may reflect behavioural differences, such as more outdoor activities, rough play, or occupational exposures in males, which can cause skin abrasions that allow bacteria to enter as supported by **Agbanyo** et al. 2025 [21]. The same applies to the age distribution of yaws patients, where our data corroborates **Boock** *et al.,* 2017 [16] finding, where 84% of patients were aged 5–14 years.

In Côte d’Ivoire, most patients in our study were located within 5 km of a health centre, and 67% of the population benefits from such proximity, according to a 2018 government report **Kra** et **Schmidt**-**Sane** 2021 [22]. This level of healthcare access may contribute to the low prevalence of yaws observed, as early detection and treatment likely reduce transmission. However, other factors, such as environmental conditions, socio-economic status, and ongoing control efforts, may also play significant roles.

Another explanation of the low prevalence of yaws could also be the fact that of the 15 districts investigated, five (Vavoua, Bangolo, Korhogo, Minignan and Nassian) had previously been treated in 2021 for trachoma using azithromycin and in these districts the prevalence of yaws was very low. Korhogo, Minignan and Nassian are situated in the dry and northern part of Côte d’Ivoire

The absence of a molecular diagnostic test (PCR) in our study could also explain the low prevalence of yaws. This is emphasized by **Marks**, **Vahi**, *et al.,* 2015 [23] who support that in many settings, up to 50% of possible yaws lesions are not confirmed serologically. Molecular diagnostics are therefore a central component of the eradication strategy **Marks**, **Mitjà**, et al. 2015 [24].

During the investigation for yaws, other skin lesions were documented. These lesions included other cutaneous NTDs from other common dermatoses. The results show the co-endemic nature of NTDs in the same communities, as mentioned by **Koffi** et al. 2020 [25] in their approach to combating NTDs in Côte d’Ivoire. In addition, the study showed that other skin lesions are prevalent in the same communities and could constitute a health problem for the population due to the number of people affected. Indeed, for 65 772 children with no yaws lesions, 21% cases of traumatic and 34.4% of mycotic (Dermatophytosis, Pityriasis versicolor) lesions were reported. For **Yotsu** 2018 [26] the sheer number of these lesions found in the same communities should argue in favour of managing them as well as skin NTDs.

Compared to a previous study on yaws in Côte d’Ivoire conducted by **Touré** *et al.,* 2007, the prevalence found in our study was lower than the prevalence of 5 per 1000 that they found. Additionally, the yaws diagnosis during their investigation was based on clinical lesions only. In our study, we employed serological tests. They also found that most of the patients were male (91%). A great difference remained in the target population: while we considered children aged under 15 years, they included patients aged 15 years and older; only just 27% of diagnosed patients had received medical treatment at the time of the study, whereas we treated all the patients enrolled in our study.

## Limitations of the study

The study had several limitations:

- For financial and logistical reasons, the study was carried out in a limited number of districts (15/113). The sampling was not representative; it was purposive. This poses a problem of extrapolating the data to all 113 districts in Côte d’Ivoire.
- Samples were not collected from all the positive cases for PCR testing during the study. Only ulcerative lesions were collected. Macula and keratosis could not be collected. For those collected, PCR results were not available to confirm serological diagnosis.
- Latent yaws can affect the study’s conclusions insofar as a large proportion of patients suspected to have active early yaws are found to have latent yaws (reactive serological tests). Diagnosis for the current skin lesion is often different from Yaws when molecular tests are used.
- According to **Engelkens** *et al.,* 1999 [27] patients with primary and secondary yaws may pass into a period of latency after resolution of clinical signs. So, it is not known how many patients are infected without developing clinical disease. All this could have an important impact on our conclusions considering that all these patients with latent yaws and no clinical signs may have been ignored.
- Adults were not tested for yaws, but yaws may still occur among adults when infected by children, especially in remote villages where health services are lacking, and environmental sanitation and personal hygiene are poor. Latent yaws among close contacts, whether children or adults, have been important reservoirs of infection.
- As an impact, a failure to identify contacts of infected individuals, inadequate treatment of latent yaws could have led to the eventual failure of the elimination strategy supported by **Antal** *et al.,* 2002.
- The investigation was limited to children in primary schools and rural areas only. Apart from 2 secondary schools close to rural area; secondary schools were not included because most of them are in urban area where yaws prevalence is low. These establishments may house children aged under 15 years.
- There might be an association of water quality and infection status but in the present study no association or even correlation was analysed and described with yaws or skin diseases.

## Conclusion

The yaws survey is an initiative of the Ministry of Health aimed at determining the degree of endemicity of the disease in Côte d’Ivoire. The investigation took place with a strong commitment from the community. The study achieved its objectives by identifying districts endemic to yaws. The activity was carried out according to the planned methodology. Data collection showed that yaws is endemic in Côte d’Ivoire, with a very low and variable prevalence. This prevalence is higher in forested areas with high rainfall consistent with the epidemiology of the disease. The results provide guidance for developing a plan to eradicate yaws in Côte d’Ivoire. Therefore, the next study should focus on a comprehensive survey of all Côte d’Ivoire districts and enlarging the target to random sampling of the entire population to provide a true picture of Yaws situation in the country for a global plan to eradicate yaws. Also, the next study should be couple with drug mass administration campaign and set up surveillance using PCR to improve diagnostic for eradication of yaws.

## Supporting information

S1 File Courrier DGS. (PDF)

S2 File Number of yaws suspected cases notified in the targeted districts over the last 10 years in Côte d’Ivoire (Côte d’Ivoire Annual Report on the Health Situation | GHDx (healthdata.org). (DOCX)

S3 File Results of positive yaws testing. (XLS)
