# Supplementary material for: Yaws active case detection surveys in 15 districts of Cote d’Ivoire
Source: PLoS One. 2026 May 28;21(5):e0348510. doi: 10.1371/journal.pone.0348510 (PMC13218476; doi:10.1371/journal.pone.0348510)
Supplement: S1 File — (PDF) [file pone.0348510.s001.pdf]

MINISTERE DE LA SANTE,
DE L'HYGIENE PUBLIQUE ET DE LA
COUVERTURE MALADIE UNIVERSELLE

DIRECTION GENERALE DE LA SANTE

PROGRAMME NATIONAL DE
LUTTE CONTRE L'ULCERE DE BURULI

REPUBLIQUE DE CÔTE D'IVOIRE
Union – Discipline – Travail

Abidjan, le 28 AVR. 2021

N°390 /MSHPCMU/DGS/PNLUB/kat

À

Messieurs
Les Directeurs Régionaux de la Santé, de l'Hygiène Publique et de la Couverture Maladie Universelle du :

Folon, Bafing, Bounkani, Gontougo, Bélier, Guenon, Haut-Sassandra, Gbokiè, Sud-comoé, Indenie-Djuablin, Agneby-Tiassa

Objet : projet d'investigation rapide du pian dans 15 districts sanitaires en Côte d'ivoire.

En vue d'atteindre la cible de l'éradication du pian d'ici 2030 tel que définie par l'OMS, le Ministère de la Santé, de l'Hygiène Publique et de la Couverture Maladie Universelle (MSHPCMU) mettra en œuvre à travers le Programme National de Lutte contre l'Ulcère de Buruli (PNLUB), une investigation rapide de cette maladie dans les districts sanitaires de : ***Minignan, Touba, Korhogo, Nassian, Bondoukou, Tanda, Didievi, Tiebissou, Vavoua, Bangolo, Sassandra, Issia, Adiaké, Abengourou, Agboville.***

Le projet dénommé « ***Proposition pour l'évaluation rapide de l'incidence du pian dans 15 districts sanitaire sentinelle en Côte d'Ivoire*** » se déroulera grâce à l'appui technique et financier de **la Fondation Anesvad.**

A cet effet, je vous demande de prendre toutes les dispositions nécessaires pour sa mise en œuvre conformément au chronogramme établit.

Le Directeur Général de la Santé

Professeur Mamadou SAMBA

Ampliation : DDSHPHCMU concernés
PJ : chronogramme de l'activité

MINISTRY OF HEALTH PUBLIC HYGIENE AND UNIVERSAL HEALTH COVERAGE

DIRECTORATE GENERAL FOR HEALTH

NATIONAL BURULI ULCER CONTROL PROGRAMME

REPUBLIC OF CÔTE D'IVOIRE

Union - Discipline - Labor

N° **1390**/MSHPCMU/DGS/PNLUB/kaf

Abidjan 28 April 2021

To
The Regional Directors of Health, Public Hygiene and universal health coverage of :
Folon, Bafing, Boukani, Gontougo,
Bélier Guémon, Haut-Sassandra,
Gboklé, Sud-Comoé, Indénie-Djuablin,
Agneby-Tiassa

Subject: Yaws investigation project
In 15 health districts in Côte d'Ivoire

In order to achieve the WHO target of eradicating yaws by 2030, the Ministry of Health, Public Hygiene and Universal Coverage (MSHPCMU), through the National Buruli Ulcer Control Programme (PNLUB), will implement a rapid investigation of this disease in the following health districts: **Minignan, Touba, Korhogo, Nassian, Bondoukou, Tanda, Didievi, Tiebissou, Vavoua, Bangolo, Sassandra, Issia, Adiaké, Abendourou and Agboville.**

The project called « **Proposal for a rapid assessment of the incidence of yaws in 15 sentinel districts in Côte d'Ivoire**» will take place thanks to the financial and technical support of the **Anesvad Foundation.**

To this end, I would ask you to take all the necessary steps for its implementation in accordance with the established timetable.

The Director General of Health

Professor Mamadou Samba

Ampliation: Health district targeted

Attached: activity timetable
