# Supplementary material for: Yaws active case detection surveys in 15 districts of Cote d’Ivoire
Source: PLoS One. 2026 May 28;21(5):e0348510. doi: 10.1371/journal.pone.0348510 (PMC13218476; doi:10.1371/journal.pone.0348510)
Supplement: S2 File — (DOCX) [file pone.0348510.s002.docx]

Table: Number of suspected yaws cases notified by district over the last 10 years

| **ZONES** | **TARGET DISTRICTS** | **NUMBER OF SUSPECTED YAWS CASES NOTIFIED OVER THE LAST 10 YEARS** |
| --- | --- | --- |
| Northwest | Minignan | 0 |
|  | Touba | 51 |
|  | Korhogo | 275 |
| North-East | Nassian | 0 |
|  | Bondoukou | 242 |
|  | Tanda | 1642 |
| Centre | Didievi | 30 |
|  | Tiebissou | 608 |
|  | Vavoua | 929 |
| South West | Bangolo | 207 |
|  | Sassandra | 1012 |
|  | Issia | 1853 |
| South-East | Adiaké | 212 |
|  | Abengourou | 987 |
|  | Agboville | 1412 |
